# High Throughput Phenotypic Selection of *Mycobacterium tuberculosis* Mutants with Impaired Resistance to Reactive Oxygen Species Identifies Genes Important for Intracellular Growth

**DOI:** 10.1371/journal.pone.0053486

**Published:** 2013-01-08

**Authors:** Olga Mestre, Raquel Hurtado-Ortiz, Tiago Dos Vultos, Amine Namouchi, Mena Cimino, Madalena Pimentel, Olivier Neyrolles, Brigitte Gicquel

**Affiliations:** 1 Unité de Génétique Mycobactérienne, Institut Pasteur, Paris, France; 2 Centro de Patogénese Molecular, Unidade dos Retrovírus e Infecções Associadas, Faculdade de Farmácia, Universidade de Lisboa, Lisboa, Portugal; 3 Centre National de la Recherche Scientifique, Institut de Pharmacologie et de Biologie Structurale, Toulouse, France; 4 Université de Toulouse, Université Paul Sabatier, Institut de Pharmacologie et de Biologie Structurale, Toulouse, France; Tulane University, United States of America

## Abstract

*Mycobacterium tuberculosis* has the remarkable capacity to survive within the hostile environment of the macrophage, and to resist potent antibacterial molecules such as reactive oxygen species (ROS). Thus, understanding mycobacterial resistance mechanisms against ROS may contribute to the development of new anti-tuberculosis therapies. Here we identified genes involved in such mechanisms by screening a high-density transposon mutant library, and we show that several of them are involved in the intracellular lifestyle of the pathogen. Many of these genes were found to play a part in cell envelope functions, further strengthening the important role of the mycobacterial cell envelope in protection against aggressions such as the ones caused by ROS inside host cells.

## Introduction

*Mycobacterium tuberculosis*, the causative agent of tuberculosis, infects 1/3 of the world’s population and is responsible for around 1.4 million deaths each year (WHO). The success of this pathogen relies in part on its ability to thrive inside macrophages. This is due to the many strategies it has evolved to resist killing by professional phagocytes, including phagosome maturation arrest [1] and resistance to strong antimicrobial molecules produced by phagocytes, such as reactive nitrogen species (RNS) and reactive oxygen species (ROS) [2]. Therefore, defence mechanisms against RNS and ROS are important for *M. tuberculosis* pathogenicity. Understanding the molecular basis of such mechanisms may provide clues for the development of target-specific drugs or effective attenuated strain-based vaccine candidates.

The generation of ROS is initiated by phagocyte NADPH oxidase (Phox) that produces superoxide anions. These anions have a low bactericidal potential, however they rapidly dismutate into H_2_O_2_, which can produce hydroxyl radicals when reacting with iron through what is known as the Fenton reaction. These molecules are highly reactive agents, damaging proteins, lipids, carbohydrates and nucleic acids [3]. The biological importance of ROS is clearly demonstrated in patients with chronic granulomatous disease (CGD), which is characterized by a total absence or low level of ROS due to deficiencies in Phox-encoding genes. These individuals are highly susceptible to bacterial, fungal and yeast infections [3], and increasing numbers of cases of mycobacterial diseases, including tuberculosis, are being diagnosed in these patients [4]. Nonetheless the significance of ROS in host defence against *M. tuberculosis* remains controversial. Despite the early demonstration that ROS were mycobactericidal [5], years later it was shown that *M. tuberculosis* could be resistant to killing by oxidative stress in macrophages [6]. *M. tuberculosis* genes showing similarities with genes known to be involved in ROS resistance in different bacterial species were identified [7], [8]. However, transcriptomic analysis showed that many of these genes are not induced in oxidative conditions [9]. This may be explained by a relatively high and constitutive expression level of such genes [10] together with an absence of the classical oxidative stress response regulators SoxR and OxyR in *M. tuberculosis* [7]. This suggests that bacilli are continuously primed for oxidative stress defense, revealing once again the importance of this kind of responses in *M. tuberculosis*. Still, evidence for a role in defence against ROS mainly comes from a *M. tuberculosis katG* mutant, with an alteration of both catalase and peroxidase activities, which was attenuated in wild-type mice but grew normally in NADPH oxidase-deficient mice, which are unable to produce ROS [11].

The construction of transposon mutant libraries in mycobacteria has enabled the newly identification and study of several potential virulence factors [12]–[14]. So far, different screening-based approaches using mutant libraries have been conducted to identify *M. tuberculosis* mutants sensitive to acidic [15] and nitric [16] stresses. With the aim to identify and investigate genes required for resistance against ROS in *M. tuberculosis*, we screened, for the first time, a transposon mutant library to identify mutants with impaired ability to resist oxidative stress.

## Results and Discussion

### Screening for Mutants with Increased Susceptibility to Oxidative Stress

**Figure 1:**
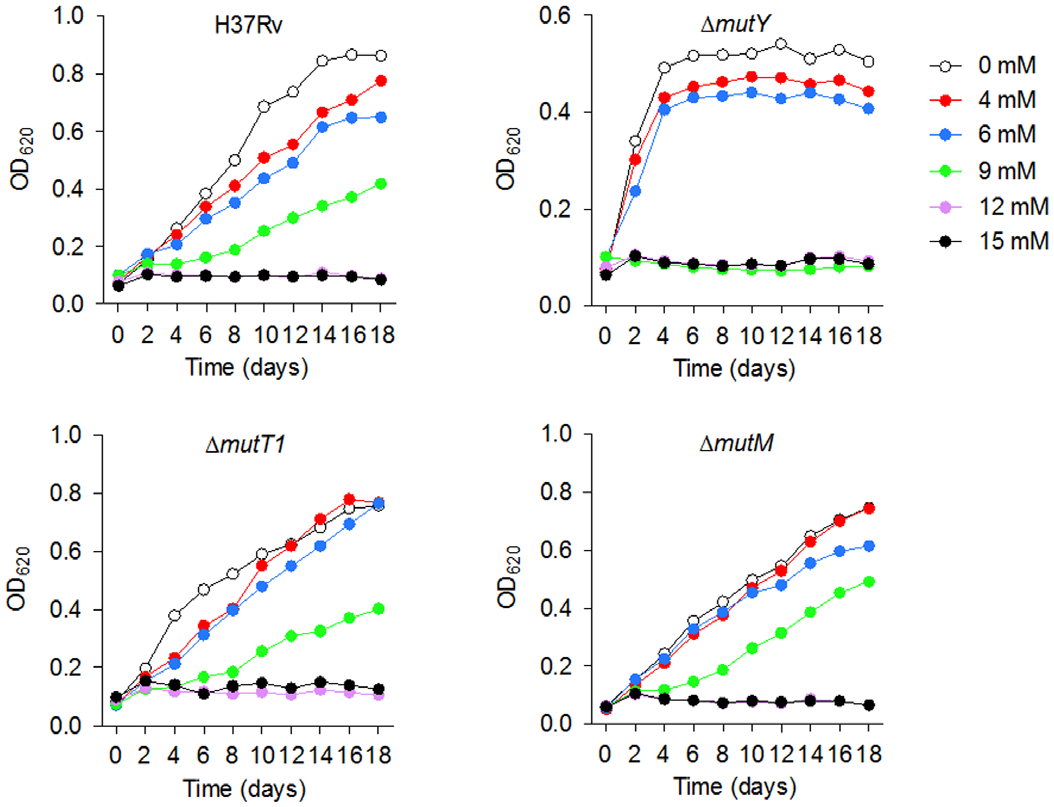
Growth Curves of H37Rv, Δ*mutT1*, Δ*mutM* and Δ*mutY* in increasing concentrations of hydrogen peroxide. Data represent means of triplicates in a representative experiment.

In order to optimize the conditions to be used to screen a mutant library and search for mutants with increased susceptibility to H_2_O_2_, we tested a small set of DNA repair mutant strains. These were knockout mutants in *mutT1*, *mutM*, and *mutY* genes, involved in handling with oxidized guanine, an important DNA lesion caused by ROS [17]. Analysis of growth rate of these strains in the presence of various concentrations of H_2_O_2_ (Figure 1) showed that the *mutY* mutant had an increased susceptibility to H_2_O_2_ as bacterial growth was totally inhibited in the presence of 9 mM H_2_O_2_ (Figure 1B), while the wild-type as well as the *mutT1* and *mutM* mutant strains were still able to grow in this condition (Figure 1A, C and D). The *mutY* mutant presented a slight growth defect in the absence of H_2_O_2_ that could also be due to oxidative stress caused by endogenous ROS resulting from aerobic respiration [18], [19]. These results suggest that MutY is important for the survival of *M. tuberculosis* under oxidative stress conditions, and allowed us to define the conditions to identify sensitive mutants to H_2_O_2_ The observed difference between these mutants might be explained by the fact that *M. tuberculosis* possesses at least four copies of *mutM* and *mutT* homologues, while a single *mutY* homolog has been annotated in mycobacterial genomes [8].

We next screened a transposon mutant library constructed in a virulent *M. tuberculosis* clinical isolate (GC1237) of the Beijing/W family [20]. Sensitive mutants were identified by comparing their OD reading values to the ones of wild-type strain, after 3 weeks of growth in 96-well plates in the presence of 9 mM H_2_O_2_. The analysis of approximately 6000 mutants allowed the selection of 18 H_2_O_2_ sensitive mutants corresponding to 11 different genes and two intergenic regions identified by sequencing of the transposon insertion sites after inverse PCR (Table 1). No significant growth defect was observed for these mutants in the absence of H_2_O_2_, indicating that their sensitivity is specific to H_2_O_2_ under the conditions used (Table 1).

**Table 1:** List of identified sensitive mutants to oxidative stress using hydrogen peroxide.

Mutantid	Gene (transposon insertion site)	Product Putative function^a^		Growth Index^b^	Growth Index H_2_O_2_^c^	Reference
118B7	Rv0305c (2701∶2702)	PPE6Unknown	1	0,99	0.35	This study
118F3	Rv0986 (430∶431)	ABC transporterThought to be involved in active transport of adhesioncomponent across the membrane	1	1,04	0.24	[22], [23], [34]
100E6	Rv1507c (591∶592)	Conserved hypothetical proteinUnknown	3	0.95	0.36	[20]
96F9	Rv1509 (637∶638)	Hypothetical proteinUnknown	5	0,89	0.34	This study
100C5	Rv1548c (1125∶1126)	PPE21Unknown	1	0.85	0.38	[36]
100D1	Rv2337c (−41)*	Hypothetical proteinUnknown	5	1,05	0.15	This study
98F4	Rv2339(1290∶1291)	Transmembrane transport protein MmpL9Thought to be involved in fatty acid transport	1	0.80	0.13	[38]–[40]
100D7	Rv2339 (957∶958)	Transmembrane transport protein MmpL9 1Thought to be involved in fatty acid transport		1,02	0.31	[38]–[40]
117C10	Rv2339 (812∶813)	Transmembrane transport protein MmpL9Thought to be involved in fatty acid transport	1	0,95	0.23	[38]–[40]
98G4	Rv2480c-Rv2481c (2786248∶2786249)	Possible transposase for insertion sequence element IS6110 (fragment)-Hypothetical protein		0.76	0.14	–
115C4	Rv3112 (49∶50)	Molybdenum cofactor biosynthesis proteinMoaD1 Involved in molybdenum cofactor biosynthesis	2	0,92	0.38	[20]
104D8	Rv3343c (2758∶2759)	PPE54Unknown	1	1.13	0.31	[20], [36]
115C10	Rv3343c (5907∶5908)	PPE54Unknown	1	1,01	0.32	[20], [36]
99G11	Rv3350c (1441∶1442)	PPE56Unknown	1	0.93	0.23	[36]
115B9	Rv3350c (5059∶5060)	PPE56Unknown	1	1,03	0.27	[36]
115C9	RV3350c (5059∶5060)	PPE56Unknown	1	0,98	0.30	[36]
106H11	Rv3430c (1049∶1050)	Possible transposaseInvolved in the transposition of the insertion sequence IS1540	4	0,75	0.20	[33]
111E12	Rv3594 (774∶775)	Conserved hypothetical proteinUnknown	3	0,74	0.09	This study

a Annotations are from tuberculist http://genolist.pasteur.fr/TubercuList/.

b, c Growth index is the ratio of the optical density value of the mutant to that of the wild-type strain after 3 weeks growth in the absence (b) or presence (c) of H_2_O_2_ 9 mM. Mutants with a growth index below 0.4 were selected as sensitive to H_2_O_2_.

* Tn inserted 41 bp upstream the gene start codon.

1 Cell envelope associated function, 2 Intermediary metabolism and respiration, 3 Conserved hypotheticals, 4 Insertion sequences and phages, 5 Unknown.

### Sensitive Mutants to Oxidative Stress are Affected in Cell Envelope Functions

Interestingly, many of the sensitive mutants to oxidative stress identified harboured defects in genes involved in *M. tuberculosis* cell envelope functions (Table 1), including several *ppe* genes, that code for cell surface proteins present almost exclusively in pathogenic mycobacteria and thought to be involved in virulence [21], as well as an ABC transporter (Rv0986) implicated either in the transport of an adherence factor or in mycobacterial cell wall architecture and integrity [22], [23].

Strikingly, we could independently isolate three different mutants in the *mmpL9* gene, which suggests an important role for MmpL9 in *M. tuberculosis* response to oxidative stress (Table 1). MmpLs (mycobacterial membrane protein large) are thought to be involved in lipid synthesis and/or transport [24] and this substrate specificity seems to be dictated, in part, by their interaction with particular cytosolic enzymes, as shown for MmpL7 [25]. Some MmpLs, such as MmpL3, might even be involved in more than one physiological process in *M. tuberculosis* [26]. MmpL9 could be thus involved in the transport of lipids and/or other kind of substances related to detoxification processes responsible to maintain an oxidative balance within the cell.

Some genes with no apparent known function were also identified in our screen (Table 1). It seems though, that *rv1507c* belongs to a cluster of genes organized in an operon (*rv1503c*-*rv1507c*) involved in di-O-acyl-trehalose (DAT) lipid synthesis [20]. The *rv1509* gene seems to form an operon along with *rv1508a.* BLAST analysis identified methyltransferase domains in the Rv1509 protein. Rv1508a shows homology with GDP-D-Mannose-Dehydratase (GMD). GMD is involved in the production of L-fucose from GDP-D-mannose. Fucose is part of phenolic glycolipids (PGL) found in Beijing strains. However other GMD has been identified that seems to be already involved in this pathway [27]. Thus, whether Rv1508a along with Rv1509 are involved in lipid synthesis is unclear. Rv3594, another protein with unknown function found in our screen, shows similarities with an N-acetylmuramoyl-L-alanine-amidase, a peptidoglycan hydrolase. Peptidoglycan hydrolases participate in bacterial cell wall growth, in its regulation and also in different lysis phenomena [28].

We also identified a few genes not associated with *M. tuberculosis* cell envelope functions. It was the case of *moaD1,* recently confirmed as involved in the biosynthesis of the molybdenum cofactor (MoCo) in *M. tuberculosis* [29]. A transcritptomic analysis shows that *moeB1*, another player in MoCo biosynthesis, is also upregulated under oxidative conditions, [30] suggesting that MoCo is important for *M. tuberculosis* to counteract oxidative stress. MoCo seems to be required for the activity of several *M. tuberculosis* enzymes that present recognizable MoCo-associated domains, including biotin sulfoxide reductase [29]. This enzyme seems important for protection from oxidative stress in *E. coli* by reducing biotin sulfoxide, the product of oxidized biotin, into biotin [31]. However, whether this enzyme is functional and whether it has the same role in *M. tuberculosis* as in *E. coli* remains to be determined.

Strikingly, a mutant in a putative transposase (Rv3430c) was also selected as sensitive to oxidative stress. A significant proportion of insertion element genes including transposases were also found to be highly induced in *M. tuberculosis* after exposure to several DNA-damaging agents, including H_2_O_2_ [32]. However, the correlation between these mobile genetic elements and oxidative stress or DNA damage/repair is not clear. This gene was also found to be important for *M. tuberculosis* survival in the lungs of non-human primates [33], suggesting a role for Rv3430c *in vivo*.

We did not identify known antioxidant genes, such as *katG* or *sodC*, during our screen. It is possible that the 6000 mutants screened may not cover the 4000 open reading frames in the genome of *M. tuberculosis* [8]. On the other hand, some of these mutants might be sensitive to endogenous ROS resulting from aerobic respiration [18], [19], which can induce *in vitro* growth defects in the absence of H_2_O_2_. In this case, they could have been excluded from the study before the screening (Materials and Methods). Indeed, an exhaustive study of *M. tuberculosis* whole genome expression in response to oxidative stress caused by H_2_O_2_ has shown that *katG*, *sodA*, *sodC*, *ahpD*, *ahpE* and *ahpC* have high basal levels of expression [10]. However, most of these are not highly induced by H_2_O_2_. In addition, these genes are also not consistently induced in *M. tuberculosis* inside host cells [9], [34]. Thus, these genes might be important to counteract low or basal levels of ROS, however, other pathways are induced when bacteria are exposed to additional levels of oxidative stress, such as those produced during phagocytic oxidative burst [18], [19]. On the other hand, *M. tuberculosis* oxidative stress response is concentration-dependent [10], as previously shown for *E. coli* [35]. Therefore, different ROS levels induce different kinds of responses. Finally, it is also possible that some of these genes have redundant functions and some mutants might not be sensitive to H_2_O_2_ but to other forms of oxidative stress.

### Quantification of Mutants Sensitivity to Oxidative Stress

**Figure 2:**
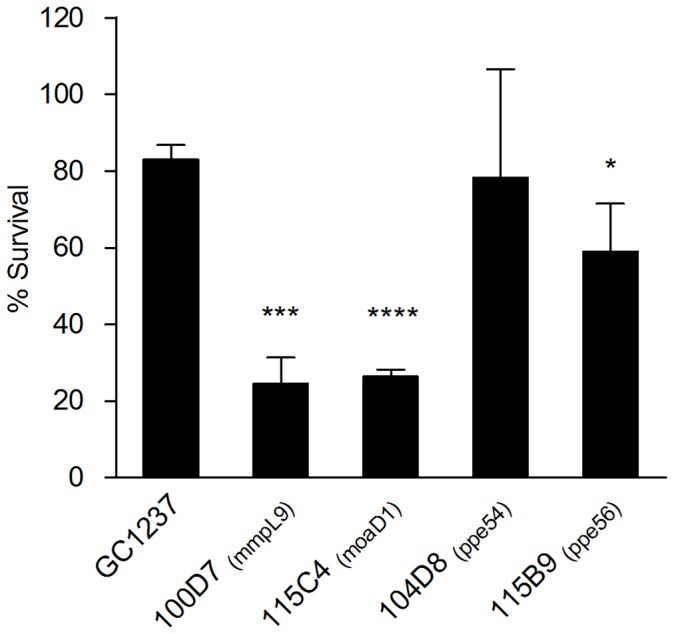
Susceptibility of transposon mutants to oxidative stress. Percentage of survival of the *M. tuberculosis* 100D7 (*mmpL9*), 115C4 (*moaD1*), 104D8 (*ppe54*), 115B9 (*ppe56*) transposon mutants and wild-type (GC1237) strains was determined by comparing bacteria treated with 9 mM of hydrogen peroxide to untreated controls. Data and error bars represent means and standard deviations of the results from triplicates in a representative experiment carried out three times with similar results. Statistically significant differences in survival of mutant strains when compared to wild-type are indicated by asterisks *P<0,05; ***P<0,001; ****P<0,0001.

The sensitive phenotype of our newly identified mutants to oxidative stress was further analyzed and quantified on the individual level for several of those mutants possibly affected in cell envelope components including two of the PPE mutants, *ppe54* (104D8) and *ppe56* (115B9), and a *mmpL9* mutant (100D7), as well as for the *moaD1* mutant (115C4), possibly implicated in other kind of resistance mechanism against oxidative stress (Table 1). This was done by analyzing the killing effect of H_2_O_2_ on bacteria. As shown in Figure 2, all mutant strains, except the *ppe54* mutant, showed an increased sensitivity to killing by H_2_O_2_ when compared to their wild-type counterpart. We observed a decrease in mutant strain survival ranging from around 40% (104D8, *ppe56* mutant) to around 80% (100D7, *mmpL9* mutant) while the killing rate for the wild-type strain was less than 20% (Figure 2). There was no difference in colony size or morphology of the mutants when compared to the wild-type strain in the absence of H_2_O_2_ (data not shown). These results confirm the sensitive phenotype of these strains to oxidative stress in the presence of H_2_O_2_ and reveal its bactericidal effects. The difference in sensitivity to H_2_O_2_ between the *ppe54* mutant and the wild-type strain was not statistically significant. This suggests that the effect of H_2_O_2_ on this strain is not bactericidal but probably only bacteriostatic as we observed growth inhibition in the presence of H_2_O_2_ during the screen (Table 1). Additionally, two different mutants carrying independent transposon insertions in the *ppe54* gene were selected (Table 1), confirming the sensitivity to oxidative stress of mutants in this gene.

### Phenotype of Sensitive Mutants to Oxidative Stress in Host Cells

Several genes identified in our screen as important for *M. tuberculosis* response to oxidative stress seem to be also important for bacterial survival inside host cells. For example, *ppe54* was identified in a screening using the same transposon mutant library, as involved in phagosome maturation arrest and was attenuated in mouse macrophages [20]. In addition, *M. tuberculosis ppe54* along with *ppe56* and *ppe21* expression was shown to be induced in IFNγ-activated macrophages [36]. The Rv0986 transporter was also previously identified as important for *M. tuberculosis* to bind and replicate inside macrophages [22], [23], as well as being involved in phagosome maturation arrest [22], [23], [34]. The *moaD1* was also associated with phagosome maturation arrest [20] and other genes involved in the MoCo biosynthetic pathway have been shown to be important for *M. tuberculosis* to parasitize macrophages [23], [30] which suggests a role for MoCo in bacterial persistence inside host cells. To further investigate this phenotype, we decided to analyze the growth in human macrophages of the *moaD1* mutant, along with the *mmpL9* mutant. MmpLs have been identified as important determinants of *M. tuberculosis* virulence [12], [13], [37]–[39] even though reports regarding *mmpL9* mutant phenotype in mice are contradictory [13], [38]–[40]. In addition, the *moaD1* and *mmpL9* mutants were found to be the most sensitive strains to killing by H_2_O_2_ (Figure 2).

**Figure 3:**
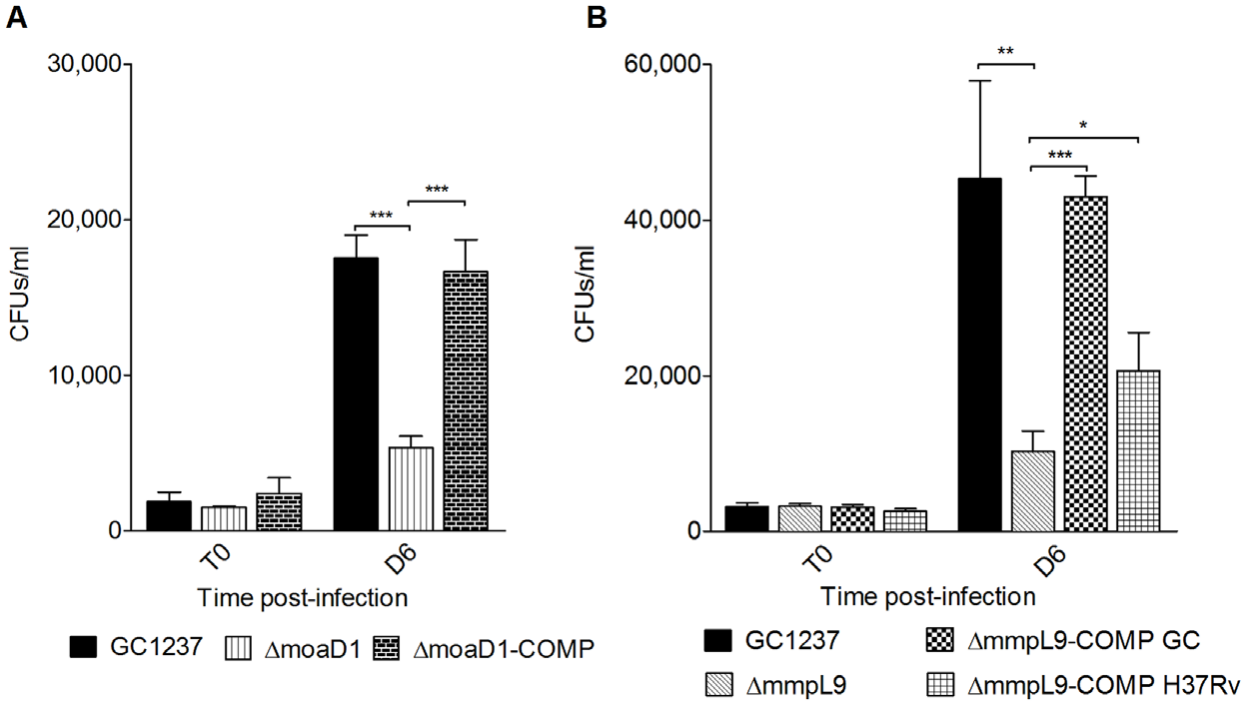
Intracellular growth of *mmpL9* and *moaD1* mutants in human macrophages. Monocyte-derived macrophages where infected with wild-type, *moaD1* (A) or *mmpL9* (B) mutant and complemented (COMP) strains at an MOI of 1∶100, bacteria per macrophage. At time point zero (0) and six days of infection, cells were lysed and bacteria were enumerated. Data and error bars represent means and standard deviations of the results from triplicates in a representative experiment carried out three times with similar results. Statistically significant differences are indicated by asterisks *P<0,05; **P<0,01;***P<0,001.

Monocyte-derived macrophages were infected with mutant, wild-type and complemented strains. We observed an impaired intracellular growth of both *moaD1* and *mmpL9* (100D7) mutants when compared to their wild-type counterpart (Figure 3A and B). At day 6 post- infection, the *moaD1* mutant showed a 4-fold increase in intracellular growth while the wild-type showed a 12-fold increase, with respect to time-point zero. Complementation of the *moaD1* mutant using an integrative vector restored the wild-type phenotype inside macrophages (Figure 3A). These results suggest that MoCo might be indeed important for bacterial growth inside macrophages. Nitrate reductase (NarGHJI) is a MoCo-enzyme in *M. tuberculosis* [29] that seems to have both nitrate respiratory [41] and assimilatory functions [42]. This is particularly relevant for intracellular bacteria, where nutrients are limited, but nitrate can be spontaneously generated from nitric oxide, produced by macrophages and would explain, at least in part, the importance of MoCo in *M. tuberculosis* survival inside host cells. Additionally, MoCo also appears to be important for *M. tuberculosis* to grow in primate lungs [33], revealing the significance of this molecule during infection *in vivo*.

Infection of human macrophages with the *mmpL9* mutant resulted in an increase of 2.5-fold while the wild-type had a 15-fold increase in CFUs, after 6 days of infection (Figure 3B). Sequence analysis of the *mmpL9* gene of the wild-type strain GC1237 revealed, when compared to H37Rv, the presence of one nucleotide deletion at position 218, which introduced a non-sense codon. To better understand the possible effects of this polymorphism we complemented the *mmpL9* mutant with *mmpL9* of either GC1237 or H37Rv. As shown in Figure 3B, complementation restored the wild-type phenotype inside macrophages using *mmpL9* of GC1237, however, it seems to be only partially restored when complemented with H37Rv *mmpL9*.

**Figure 4:**
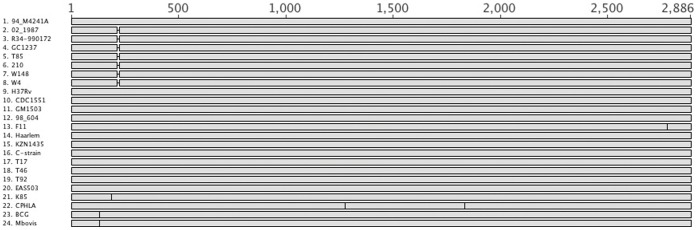
Graphical representation of the distribution of polymorphisms in *mmpL9* in several *M. tuberculosis* genomes. Polymorphisms in *mmpL9* were analyzed in 24 different *M. tuberculosis* genomes [51] that correspond to the following groups/lineages according to Gagneux *et al.* classification [52]: 1–8: Beijing/W strains; 9–16: Euro-American strains; 17–20: Indo-oceanic strains; 21–22: West-African strains. Black vertical lines correspond to SNPs. Gap correspond to deletion in position 218 identified in all modern and ancestral (2–8) but not in early ancestral (1) Beijing/W strains.

Unfortunately, we could not detect expression of either H37Rv or GC1237 *mmpL9* using Western blotting, under the control of its own or of a strong promoter (*hsp60*) and carrying a hemagglutinin tag (data not shown). This might be due to protein’s level of expression in the experimental conditions used in this study. Nonetheless, the fact that a transposon mutant had an attenuated phenotype inside macrophages that was restored after complementation with the wild-type gene (Figure 3B) suggests that the GC1237 *mmpL9* gene might be functional. Translation of *mmpL9* in GC1237 might occur due to a recoding mechanism [43], for example through translation reinitiation, in the same reading frame, from a second initiation codon, at position 259 (Figure S1). This would mean that the Beijing *mmpL9* gene is a modified protein that has acquired a new activity, which may explain the partial complementation by H37Rv *mmpL9* in macrophages. Sequence analysis in a collection of *M. tuberculosis* strains suggest that the *mmpL9* gene does not seem to accumulate more polymorphisms than the other *mmpLs* (Table 2), excluding the hypothesis of pseudogene formation but not that of an effect of this polymorphism on protein’s functions. In addition, we found this deletion to be a well conserved polymorphism, specific to all modern and ancestral Beijing/W strains, and absent in strains of any other *M. tuberculosis* family (Figure 4), suggesting that this polymorphism might contribute to the evolution of these strains by conferring some selective advantage. This is of importance because the Beijing/W family is one of the most successful groups of *M. tuberculosis* strains as it has been frequently associated with drug resistance and found to be distributed throughout the world [44].

**Table 2:** Statistical analysis of SNP density in *mmpL* genes in a collection of *M. tuberculosis* genomes.

Name	Length (bp)	SNPs	Binomial test^a^	Bonferroni correction^b^
*mmpL1*	2877	3	0,926187042	12,96661859
*mmpL2*	2907	9	0,167599855	2,346397974
*mmpL3*	2835	13	0,008366836	0,117135699
*mmpL4*	2904	6	0,565387826	7,915429562
*mmpL5*	2895	7	0,406734347	5,694280858
*mmpL6*	1194	6	0,026461116	0,370455623
*mmpL7*	2763	5	0,670824515	9,391543207
*mmpL8*	3270	9	0,270440559	3,786167821
*mmpL9*	2889	7	0,404469247	5,662569455
*mmpL10*	3009	2	0,980458231	13,72641523
*mmpL11*	2901	5	0,716449242	10,03028939
*mmpL12*	3441	11	0,128927703	1,804987847
*mmpL13a*	912	4	0,071781264	1,004937698
*mmpL13b*	1413	1	0,862276271	12,07186779
Total	36210	88		

a The binomial test was used to identify *mmpL* genes that accumulate more SNPs than expected. The *p-value* was calculated according to the number of SNPs and length of each gene and the total the number of SNPs and genes length.

b The final *p-value* was estimated after Bonferroni correction.

**Figure 5:**
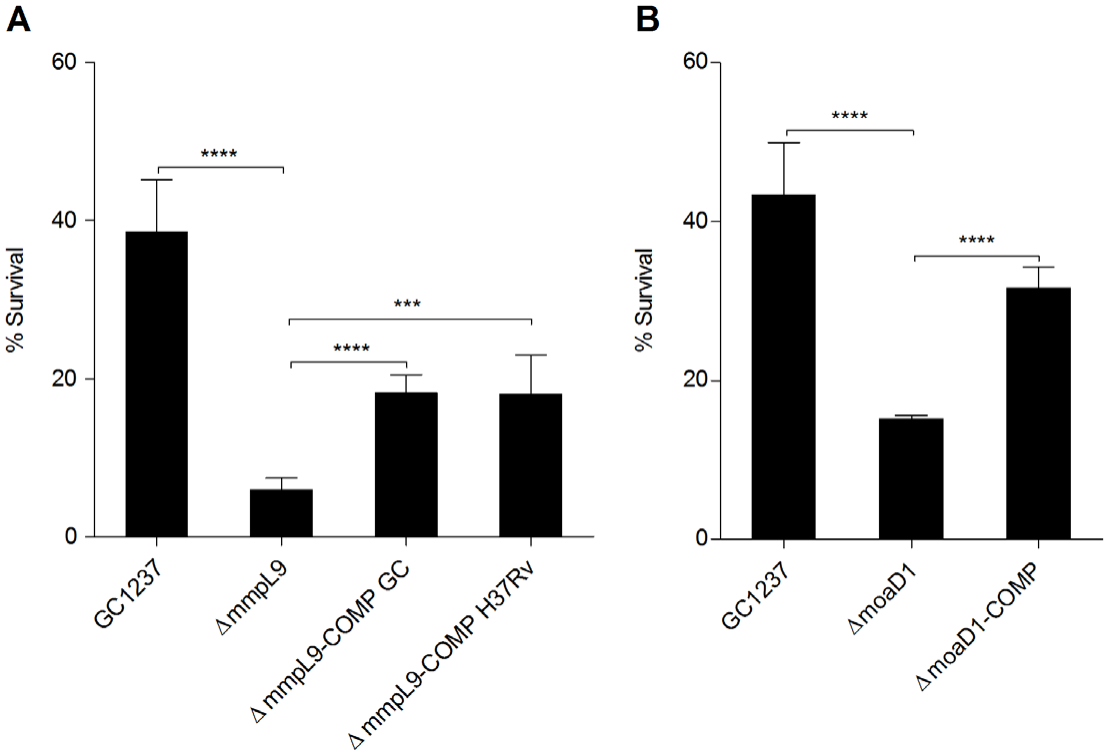
Survival of *mmpL9* and *moaD1* mutant and complemented strains exposed to hydrogen peroxide. Percentage of survival of *M. tuberculosis* wild-type (GC1237), *mmpL9* (A) and *moaD1*(B) mutant and complemented (COMP) strains was determined by comparing bacteria treated with 9 mM of hydrogen peroxide to untreated controls. Data and error bars represent means and standard deviations of the results from triplicates in a representative experiment. Statistically significant differences are indicated by asterisks ***P<0,001; ****P<0,0001.

The wild-type phenotype was partially achieved for both *moaD1* and *mmpL9* mutant complemented strains in susceptibility tests using H_2_O_2_ (Figure 5), which was unexpected because in macrophages complementation totally restored the wild-type phenotype (Figure 3). The reasons behind these observations are not understood. It could be due to differences in the fine tuning of expression of these genes because they are placed outside their normal chromosomal context. It is also likely that *mmpL9* and *moaD1* are required to protect *M. tuberculosis* against both ROS and nitric stress, as well as against acidic pH or nutrient starvation inside macrophages. Indeed, a few bacterial genes appear to exclusively participate in resistance to ROS *in vivo* [45]. We assessed the susceptibility of these mutants to acidic pH and starvation and did not notice any difference in fitness as compared to the wild-type strain (data not shown). These mutants may be susceptible to RNS or some products resulting from the combination of these stresses, such as peroxynitrite, one of the most potent natural oxidants in biological systems [7], that results from the combination of ROS with RNS. Nonetheless, the *mmpL9* and *moaD1* genes should have a function in *M. tuberculosis* oxidative stress, otherwise complementation would not have restored the wild-type phenotype at all.

### Conclusion

In this study, screening of a high density transposon mutant library identified important genes involved in *M. tuberculosis* oxidative stress response and further strengthened the important role of *M. tuberculosis* cell envelope in protection against ROS. A correlation between sensitivity to oxidative stress and survival in host cells was observed, although we cannot exclude the hypothesis that an attenuated phenotype in macrophages is not exclusively due to ROS sensitivity. Most of the genes identified during our screen have not been described in previous genetic screenings as required for *M. tuberculosis* survival in macrophages [46] or in animal models such as mice and non-human primates [33], [39], [47]. They have also not been previously reported as upregulated in *M. tuberculosis* inside host cells [9], [34]. However, we cannot exclude that the genes identified in our and in previous reports are involved in the same metabolic pathway (as observed for MoaD1), or involved in the synthesis of the same factor. For example, MmpL9 and Rv0986 are transporters of a yet unidentified substrate and some of the genes we have identified have unknown annotated functions (Table 1). Similarly, numerous genes identified during analysis of *M. tuberculosis* whole genome expression in response to oxidative stress are annotated as having unknown functions [10]. Thus, our knowledge about the molecular mechanisms used by *M. tuberculosis* to counteract oxidative stress, particularly in the context of the macrophage, is far from being complete. We also have to consider that differences regarding *M. tuberculosis* strains and host cells (mice, primary, cell-lines) employed might also explain the only modest overlap of our results with those of previous studies.

Strikingly, some of the genes we identified were found to be also involved in phagosome maturation arrest in previous studies. We hypothesize that mutants in these genes are delivered to late phagosome compartments as a consequence of being more susceptible to intracellular killing. That is, indeed, the reason why mechanisms underlying phagosome maturation arrest are difficult to characterize, because any strain impaired in intracellular survival will traffic to late endosomes as a secondary effect [1].

In conclusion, genes involved in *M. tuberculosis* oxidative stress response are important for this pathogen and seem to contribute to its intracellular growth in host cells, which make the products of these genes attractive targets for the development of new drug and vaccine candidates.

## Materials and Methods

### Bacterial Strains and Growth Conditions

*M. tuberculosis* strains GC1237 and H37Rv were grown in Middlebrook 7H9 broth (Difco) supplemented with ADC and 0,05% Tween 80, except when referred, or in 7H11 Middlebrook broth (Difco) supplemented with OADC and 0,5% glycerol for CFU analysis. Mutant strains were grown in the presence of kanamycin 20 µg/ml and complemented strains grew in the presence of both kanamycin and hygromycin 20 µg/ml and 50 µg/ml, respectively.

### Mutant Library Screening

Mutants of the *M. tuberculosis* GC1237 strain transposon library [20] were grown individually at 37°C in 96-well plates in 7H9 medium for 3 weeks. Optical density (OD) was measured, at 620 nm, and mutants which presented growth defects at this phase were excluded from subsequent analysis. For those mutants, which no significant growth defects were observed, aliquots (10–20 µl) were sub-cultured in 200 µl 7H9 medium containing 9 mM of H_2_O_2_ (Sigma-Aldrich). OD was measured two to three weeks later in a plate spectrophotometer and the ratio of the OD value of the mutant to that of the wild-type strains (growth index) was calculated. Mutants with a growth index below 0.4 were considered as sensitive to H_2_O_2_ (Table 1).

### Identification of Transposon Insertion Site

Genomic DNA from the selected transposon mutants was extracted and the exact location of the transposon insertion was determined by sequencing amplicons generated by ligation-mediated inverse PCR [13]. Briefly, 1–2 µg of DNA was digested with BssHII, a low-moderate frequency cutting restriction enzyme that doesn’t cut the transposon, followed by heat inactivation of the restriction enzyme and analysis in an agarose gel to check for complete digestion. An aliquot of this digested DNA (<0,5 ng) was diluted (1∶10) and ligated overnight at 16°C, followed by ethanol precipitation. Using these products a PCR was done to amplify the DNA regions adjacent to the transposon using *TaKaRa La Taq* DNA polymerase (TaKaRa, Shiga, Japan) with the primers corresponding to the left (Tn_L) and the right (Tn_R) (Table S1) ends of the transposon. The amplified products were sequenced using an ABI prism 3100 Genetic Analyser (Applied Biosystems) with Big Dye Terminator v3.1 cycle sequencing Kit (Perkin Elmer Applied Biosystems, Courtaboeuf, France). Sequences were mapped in *M. tuberculosis* H37Rv genome available at Tuberculist [8]. The transposon insertions within the *mmpL9* gene, *moaD1*, *ppe54* and *ppe56* were also confirmed by Multiplex-PCR using a primer that flank the site of transposon insertion (genename_F) with primers hybrizing inside the transposon (Tn_L and Tn_R2) (Table S1).

### Complementation of *mmpL9* and *moaD1* Mutants

To genetically complement the *mmpL9* mutant, the *mmpL9* gene with its putative promoter region from the wild-type strain *M. tuberculosis* GC1237 or H37Rv, was introduced in the integrative vector pMV306 conferring hygromycin resistance [48]. The complementation construct was generated by amplifying the *mmpL9* gene along with 629 bp of the upstream intergenic region from GC1237 or H37Rv chromosomal DNA using Phusion High-fidelity DNA polymerase (Finnzymes) with the primers pMV_F and pMV_R (Table S1), containing the restriction sites of XbaI and HindIII, respectively. The amplified product was gel purified, digested with XbaI and HindIII and cloned between XbaI and HindIII sites in pMV306. The resulting plasmid was sequenced to confirm that no mutations were present, and transformed into the mutant by electroporation as described [49]. Transformed bacteria were recovered using hygromicyn and kanamycin selection (50 ug/ml for both) and the presence of the wild-type gene was confirmed by PCR using primers mmpL9_F and mmpL9_R (Table S1).

The *moaD1* mutant was complemented using the pYUB412-derived cosmid [50] I528, which carries a DNA fragment covering the 3454 to 3485 Kb region of the *M. tuberculosis* H37Rv chromosome and a hygromycin resistance cassette. Bacteria were transformed and selected as described for *mmpL9* mutant complementation.

### Hydrogen Peroxide Susceptibility Assays

Bacteria were collected from culture, washed twice with PBS and clumps were dissociated by needle passages, as performed for macrophage infection. Single-cell suspensions were adjusted to approximately 1×10^6^ CFU/ml in 7H9 medium without ADC. Then, bacteria was exposed to 9 mM of H_2_O_2_ (Sigma-Aldrich, 216763) during 4 hours at 37°C. Control untreated bacteria was also included. Dilutions were then made and plated into 7H11 Middlebrook agar. CFUs were counted after 3 weeks growth at 37°C. Percentage of survival was calculated by dividing the number of CFUs treated with H_2_O_2_ by the number of untreated CFUs and multiplying by 100.

For assays in liquid medium, bacteria were grown in 7H9 medium at 37°C in the presence of different concentrations of H_2_O_2_ (Sigma-Aldrich, 216763) and OD_600_ values were measured every three days, during 2 to 3 weeks.

### Monocyte Isolation for Subsequent Macrophage Generation

Freshly collected buffy coats were obtained from healthy donors. PBMC fraction was collected using a Ficoll gradient (Ficoll-Paque™ Plus, GE Health Care 17-1440-03) and monocytes were purified by positive selection using anti-CD14 conjugated magnetic microbeads (Milteny Biotec, 130-050-20) and Quadro MACS Separation System (Milteny Biotec) according to manufacturer’s protocol. Monocytes were distributed into 6-well plates and allow to adhere in a 5% CO_2_ incubator at 37°C in 3 ml of RPMI-1640 (Sigma, R0883) containing 2 mM of L-glutamine (Gibco, 25030) supplemented with 10% of heat inactivated foetal bovine serum (PAN Biotech) and 50 ng/ml of rhM-CSF (R&D Systems, 216-MC) to differentiate into macrophages. Cultures were fed every 2 days with complete medium containing full doses of cytokine during 6 days.

Before infection, macrophages were recovered using a non-enzymatic cell dissociation solution (Sigma, 120M0859), were counted and adjusted to 2×10^5^ per well in a 24-well plates, where they were allowed to adhere.

### Macrophage Infections

Bacteria were pre-cultured in 5 ml of 7H9 Middlebrook medium with Tween at 37°C for 3 weeks. Then, bacteria were growth in 30 ml of 7H9 Middlebrook without Tween in a non-shaking incubator at 37°C for 2 weeks. Bacteria were recovered, washed two times and resuspended in 1 ml of phosphate buffered saline solution. Big clumps were disassociated by 30 passages through a 0,8×40 mm needle and small clumps by 50 passages though a 0,45×10 mm needle (3 times). The optical density was measured at 600 nm and correlated with the number of bacteria in order adjust the multiplicity of infection (MOI) used for infection (1∶100, bacteria per macrophage). The medium of blood-monocyte derived macrophages was removed and replaced with 500 ul of bacterial suspensions in RPMI-1640 (Sigma, R0883) containing 2 mM of L-glutamine (Gibco, 25030) supplemented with 10% of heat inactivated foetal bovine serum (PAN Biotech). After 4 hours of infection, medium was removed and cells were incubated with fresh medium for 6 days.

At each time point post-infection, macrophages were lysed, dilutions were made and samples were plated in 7H11 Middlebrook agar. After 3 weeks of growth at 37°C, CFUs were counted. Each infection experiment was carried out using triplicates for each condition/strain.

### Ethics Statement

Human monocytes were purified from buffy coats obtained from healthy blood donors (Etablissement Français du Sang, EFS, Rungis). All donors participating to this study had given their written consent under a EFS contract C- CPSL UNT N°09/EFS/062, following articles L1243-4 and R1243-61 of the French Public Health Code and approved by the French Ministry of Science and Technology. Supply and handling of human red cells followed the guidelines of the agreement between Institut Pasteur, EFS and the regulation of blood donation in France. As a “collection” is defined by Decree 2007-1220 as “a collection, for scientific use, of biological samples from a group of persons who have been identified and selected on the basis of clinical or biological characteristics of one or several members of the group”, and as the blood samples used in our study were not obtained from individuals that have been “identified or selected on the basis of clinical or biological characteristics”, these samples do not constitute a so-called “collection”, which waives the need for ethical approval by the ethical committee “Comité de Protection des Personnes”, in agreement with Decree 2007-1220, article R1243-63.

### Statistics

Student’s two-tailed, unpaired, *t*-test was determined using GraphPad Prism software to assess the statistical significance of results.

## Supporting Information

Figure S1 **Representation of the** ***mmpL9*** **gene in** ***M. tuberculosis*** **GC1237.** The *mmpL9* gene in *M. tuberculosis* GC1237 contains a deletion (Δ) in position 218. Reinitiaton of translation might occur from a second initiation codon situated beyond the deletion, at position 259, which could explain the attenuated phenotype in macrophages of the *mmpL9* (100D7) mutant carrying a tranposon inserted at position 957∶958.
(PDF)

Table S1 **List of oligonucleotides (5′–3′) used in this study.**
(DOCX)
