# Supplementary material for: High Throughput Phenotypic Selection of Mycobacterium tuberculosis Mutants with Impaired Resistance to Reactive Oxygen Species Identifies Genes Important for Intracellular Growth
Source: PLoS One. 2013 Jan 8;8(1):e53486. doi: 10.1371/journal.pone.0053486 (PMC3540035; doi:10.1371/journal.pone.0053486)
Supplement: Figure S1 — Representation of the mmpL9 gene in M. tuberculosis GC1237. The mmpL9 gene in M. tuberculosis GC1237 contains a deletion (Δ) in position 218. Reinitiaton of translation might occur from a second initiation codon situated beyond the deletion, at position 259, which could explain the attenuated phenotype in macrophages of the mmpL9 (100D7) mutant carrying a tranposon inserted at position 957∶958. (PDF) [file pone.0053486.s001.pdf]

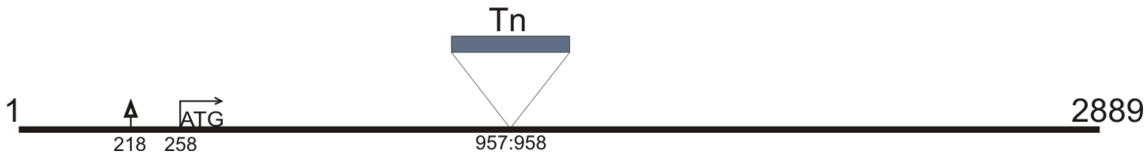

Figure S1. Representation of the *mmpL9* gene in *M. tuberculosis* GC1237. The *mmpL9* gene in *M. tuberculosis* GC1237 contains a deletion (Δ) in position 218. Reinitiaton of translation could occur from a second initiation codon situated beyond the deletion, at position 259, which could explain the attenuated phenotype in macrophages of the *mmpL9* (100D7) mutant carrying a tranposon inserted at position 957:958 (figure 3).
