# Supplementary material for: High Throughput Phenotypic Selection of Mycobacterium tuberculosis Mutants with Impaired Resistance to Reactive Oxygen Species Identifies Genes Important for Intracellular Growth
Source: PLoS One. 2013 Jan 8;8(1):e53486. doi: 10.1371/journal.pone.0053486 (PMC3540035; doi:10.1371/journal.pone.0053486)
Supplement: Table S1 — List of oligonucleotides (5′–3′) used in this study. (DOCX) [file pone.0053486.s002.docx]

**Table S1.** List of oligonucleotides (5’-3’) used in this study.

| **Primer name** | **Sequence** |
| --- | --- |
| **Tn_L** | GTCGGCCATTAGCTTCT |
| **Tn_R** | CACCACCGATCCTCAT |
| **Tn_R2** | GCACCACCGATCCTCAT |
| **mmpL9_F** | GCATCAGAACCTATGTCACCG |
| **mmpL9_R** | GATCGCATGGTCAACATCA |
| **moaD1_F** | CGCTGTCAGCGTCACTATC |
| **ppe54_F** | GGTATCGGCAATGTAGGTACTCA |
| **ppe56_F** | CATCCTCATCGGCGATA |
| **pMV_F** | ATTCTAGAGCATCATCCTCCCACGAC |
| **pMV_R** | ATAAGCTTTATCAACCCCGGTGCAGAT |
